# Motor learning is independent of effects of subthalamic deep brain stimulation on motor execution

**DOI:** 10.1093/braincomms/fcad070

**Published:** 2023-03-17

**Authors:** Christoph Muehlberg, Christopher Fricke, Mirko Wegscheider, Max Wawrzyniak, Elinor Tzvi, Dirk Winkler, Joseph Classen, Jost-Julian Rumpf

**Affiliations:** Department of Neurology, University of Leipzig Medical Center, Leipzig 04103, Germany; Department of Neurology, University of Leipzig Medical Center, Leipzig 04103, Germany; Department of Neurology, University of Leipzig Medical Center, Leipzig 04103, Germany; Department of Neurology, University of Leipzig Medical Center, Leipzig 04103, Germany; Syte Institute, Hamburg 20354, Germany; Department of Neurosurgery, University of Leipzig Medical Center, Leipzig 04103, Germany; Department of Neurology, University of Leipzig Medical Center, Leipzig 04103, Germany; Department of Neurology, University of Leipzig Medical Center, Leipzig 04103, Germany

**Keywords:** deep brain stimulation, Parkinson’s disease, motor learning, motor memory consolidation, subthalamic nucleus

## Abstract

Motor learning is defined as an improvement in performance through practice. The ability to learn new motor skills may be particularly challenged in patients with Parkinson’s disease, in whom motor execution is impaired by the disease-defining motor symptoms such as bradykinesia. Subthalamic deep brain stimulation is an effective treatment in advanced Parkinson’s disease, and its beneficial effects on Parkinsonian motor symptoms and motor execution have been widely demonstrated. Much less is known about whether deep brain stimulation directly interacts with motor learning independent of modulation of motor execution. We investigated motor sequence learning in 19 patients with Parkinson’s disease treated with subthalamic deep brain stimulation and 19 age-matched controls. In a cross-over design, patients performed an initial motor sequence training session with active and inactive stimulation, respectively (experiments separated by ≥14 days). Performance was retested after 5 min and after a 6 h consolidation interval with active stimulation. Healthy controls performed a similar experiment once. We further investigated neural correlates underlying stimulation-related effects on motor learning by exploring the association of normative subthalamic deep brain stimulation functional connectivity profiles with stimulation-related differences in performance gains during training. Pausing deep brain stimulation during initial training resulted in the inhibition of performance gains that could have been indicative of learning at the behavioural level. Task performance improved significantly during training with active deep brain stimulation, but did not reach the level of learning dynamics of healthy controls. Importantly, task performance after the 6 h consolidation interval was similar across patients with Parkinson’s disease independent of whether the initial training session had been performed with active or inactive deep brain stimulation. This indicates that early learning and subsequent consolidation were relatively intact despite severe impairments of motor execution during training with inactive deep brain stimulation. Normative connectivity analyses revealed plausible and significant connectivity of volumes of tissue activated by deep brain stimulation with several cortical areas. However, no specific connectivity profiles were associated with stimulation-dependent differences in learning during initial training. Our results show that motor learning in Parkinson’s disease is independent of modulation of motor execution by subthalamic deep brain stimulation. This indicates an important role of the subthalamic nucleus in regulating general motor execution, whereas its role in motor learning appears negligible. Because longer-term outcomes were independent of performance gains during initial training, patients with Parkinson’s disease may not need to wait for an optimal motor state to practice new motor skills.

## Teaser

Muehlberg *et al*. report that motor learning in persons with Parkinson’s disease is independent of modulation of motor execution by subthalamic deep brain stimulation during initial training. This indicates an important role of the subthalamic nucleus in regulating general motor execution, whereas its role in intrinsic motor learning seems negligible.

## Graphical Abstract

**Graphical Abstract:**
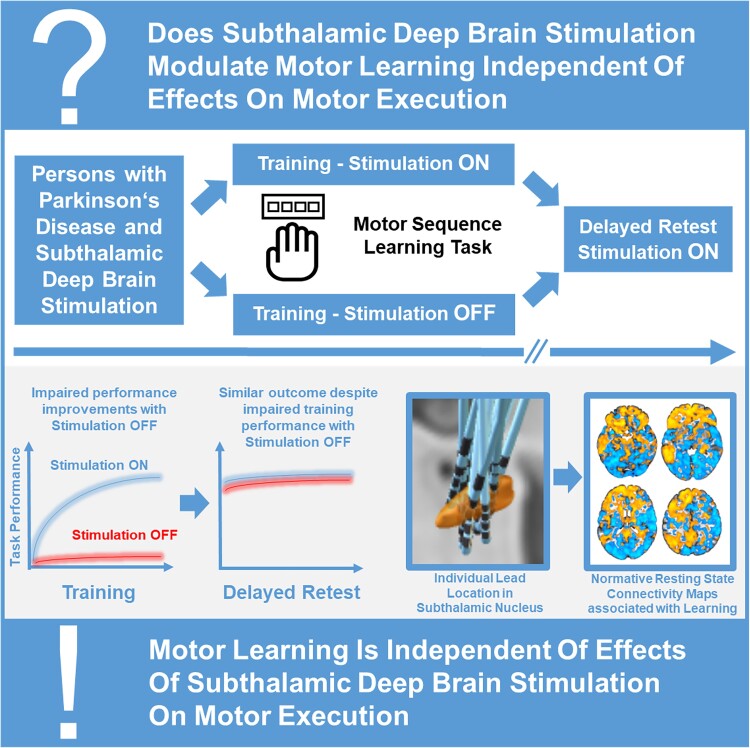

## Introduction

The ability to acquire and refine motor skills is essential to maintaining functional independence throughout life. One approach to investigate the behavioural correlates and neural mechanisms of motor learning is to apply motor sequence-learning paradigms. Typically, in these paradigms, a series of simple movements are ultimately integrated into a coherent unit through practice.^1^ Extensive research has demonstrated that motor sequence learning develops across different phases, which are sustained by distinct, however interacting, mechanisms.^1-4^ Following initial training-induced motor memory formation, the acquired motor memory trace undergoes a consolidation process in the absence of further practice, during which the initially labile motor memory is transformed into a more stable representation.^1,5,6^ Initial learning is mediated by dynamic interactions within a widespread cortical and subcortical network including the primary motor cortex, the supplementary motor area, premotor, and prefrontal cortices, basal ganglia, and cerebellum.^3,7-10^ Offline consolidation, however, is mainly regulated by cortico-striatal and cortico-hippocampal networks.^10-12^ Since motor skill acquisition relies on practice, it may be impeded by conditions associated with impaired motor execution. This is the case in Parkinson’s disease (PD), in which motor execution becomes increasingly impaired with disease progression due to worsening of characteristic motor symptoms such as bradykinesia and rigidity. The disease-defining motor symptoms are caused by the loss of nigro-striatal dopaminergic projections, resulting in a depletion of striatal dopamine.^13^ Given the striatal dysfunction in PD,^14,15^ and the important role of cortico-striatal interactions in initial online learning and offline consolidation,^5,9-12^ one would expect PD to impair motor learning. Previous research that primarily investigated early online motor learning yielded inconsistent results regarding whether motor learning is impaired in PD.^16-23^ Even less is known about whether offline motor memory consolidation is affected by the disease. The scarce evidence, however, indicates that consolidation in PD is comparable to that of age-matched healthy controls both overnight and over the day.^22,24,25^

In recent years, subthalamic deep brain stimulation (DBS) has become an established and proven effective treatment for patients with PD suffering from motor complications related to fluctuations in the efficacy of dopaminergic medication.^26,27^ Despite extensive research demonstrating that DBS improves Parkinsonian motor symptoms, much less is known about the potential effects of DBS on motor learning. A recent study by de Almeida Marcelino *et al*.^28^ reported that early motor skill acquisition was impaired in PD (when DBS was inactive) but could be substantially enhanced by DBS almost up to the level of age-matched healthy controls. However, withdrawal of DBS, by design, leads to substantial worsening of PD motor symptoms, which limits motor execution and, consequently, contaminates the range of potential training-induced performance gains. It is thus difficult to disentangle the effects of DBS on motor learning from effects on motor execution during practising. Similar limitations may apply to a smaller sample size study,^29^ which also reported a relative improvement in motor learning with active DBS compared with learning under inactive DBS across a single training session. It is generally believed that online and offline motor learning processes strongly interact and that motor memory consolidation already starts during practice.^30-36^ Because consolidation appears to be intact in PD, delayed retesting of motor performance after an offline consolidation interval may represent a more reliable indicator of how much was learned during initial training independent of motor execution at that time.

The current study aimed to address the question of whether subthalamic DBS specifically interacts with motor learning or whether the effects of DBS are limited to the facilitation of motor execution in PD. This distinction is essential as conditions that affect performance do not necessarily have to affect learning.^37^ In two separate experiments, PD patients performed initial motor sequence learning with active and inactive DBS, respectively. The primary outcome, however, was motor sequence performance after an offline consolidation interval of 6 h. This design, on the one hand, allowed us to separate the effects of DBS on motor learning from those on motor execution. On the other hand, it allowed us to investigate the interaction of DBS with online motor memory formation and subsequent offline consolidation. We also explored normative functional connectivity networks underlying DBS-associated differences in motor learning and Parkinsonian motor symptom improvements. The results provide new insights into motor skill acquisition in PD and the effects of subthalamic DBS on motor learning and motor execution.

## Materials and methods

The study protocol was approved by the local Ethics Committee (registration code: 277/20-ek) and was registered in the German Clinical Trials Register (6 August 2020; DRKS00022297).

### Participants

Forty individuals [21 PD patients treated with subthalamic DBS: mean age, 58.1 ± 7.4 years, 7 females; 19 healthy controls (HCs), 59.4 ± 6.6 years, 6 females] were recruited for the study between September 2020 and March 2022. PD patients were recruited from the movement disorders outpatient clinic at the Department of Neurology, Leipzig University Medical Center. Age-matched HCs without PD either were spouses of the recruited PD patients or recruited via local announcements. All participants were naive to the motor sequence-learning task. We excluded subjects with a history of alcohol/drug abuse, other neurological diseases than PD, acute psychosis and any severe internal or orthopaedic conditions that could have interfered with task performance. All participating individuals demonstrated no relevant cognitive impairment as assessed by the Montreal Cognitive Assessment (MoCA,^38^ exclusion cut-off <18) and no relevant depressive symptoms according to the short version of Beck’s Depression Inventory^39^ (BDI, exclusion cut-off >19). According to the Edinburgh Handedness Inventory,^40^ all participants, except one PD patient and two HCs who were classified as ambidextrous, were right-handed. PD motor symptoms were assessed by applying the motor section of the Movement Disorder Society Unified Parkinson’s Disease Rating Scale (MDS-UPDRS-III^41^).

### Experimental procedure

**Figure 1:**
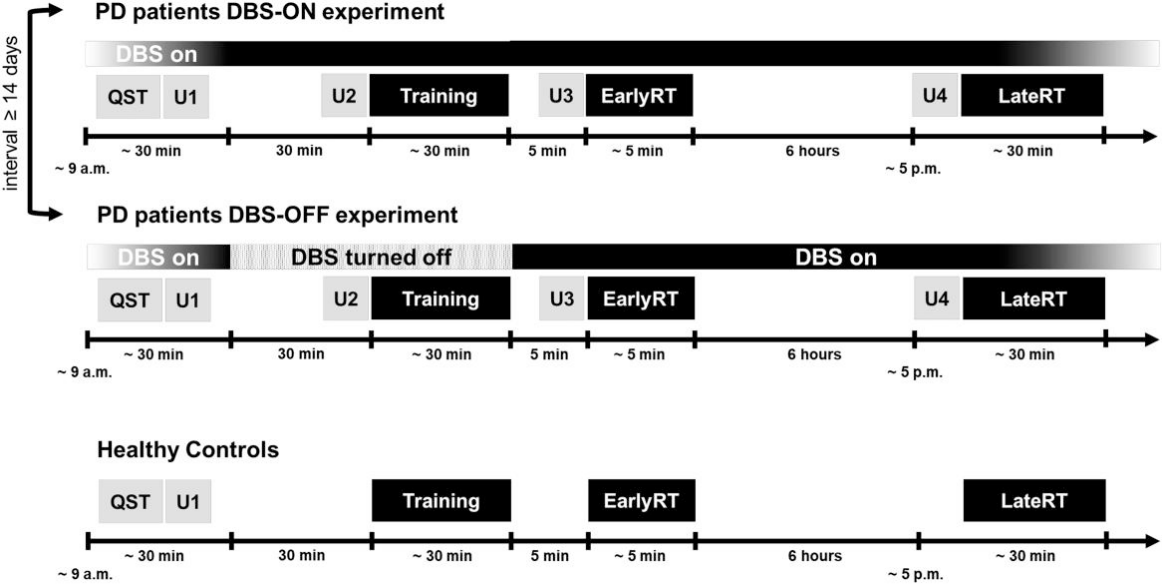
**Experimental design.** PD patients took part in two separate experiments (DBS-ON and DBS-OFF experiments) corresponding to active DBS and inactive DBS, respectively, during initial motor training. Age-matched healthy controls performed a similar experimental session once. Following the completion of questionnaires (QST), each experiment consisted of an initial motor sequence training session (Training), an EarlyRT 5 min after termination of Training, and a LateRT after an additional interval of 6 h. Parkinsonian motor symptoms according to the MDS-UPDRS-III were assessed at baseline (U1), before the initial Training session (pre-Training, U2), before early retesting (pre-EarlyRT, U3) and before late retesting (pre-LateRT, U4).

In a cross-over design, PD patients participated in two experiments separated by an interval of at least 14 days. Each experiment consisted of a motor sequence training session in the morning (Training), an early retest of motor sequence performance 5 min after termination of the training session (EarlyRT), and a late retest after a further interval of 6 h (LateRT). Experiments differed with respect to the DBS condition during the initial training session. In the DBS-OFF experiment, DBS was switched off 30 min before the onset of the training session, and participants performed the training session under inactive DBS. Immediately after the termination of the training session, DBS was turned back on so that EarlyRT and LateRT were executed under active DBS. In the DBS-ON experiment, DBS remained active all the time. PD motor symptoms (MDS-UPDRS-III) were assessed at baseline, immediately before the training session (pre-Training), and before early (pre-EarlyRT) and late retesting (pre-LateRT). Due to the narrow time window between the end of Training and EarlyRT, assessment of motor symptoms before EarlyRT consisted of only the right upper extremity subscore of the MDS-UPDRS-III (rUES), which was condensed to the items that are specifically relevant for right-hand function (i.e. ‘rigidity’, ‘finger tapping’, ‘hand movements’, ‘pronation-supination movements’, range of 0–16). No modifications were made to the regular dopaminergic PD medication for either one of the experiments. Healthy controls performed only one but otherwise identical experiment (Fig. 1). Participants were assigned to a specific combination of the order of experiments (DBS-ON/DBS-OFF experiment, PD patients only) and applied motor sequences (Seq1/Seq2) via predefined lists in the random order in which they were recruited. This ensured that conditions were balanced across experiments in PD patients and HCs.

### Motor learning task

Motor sequence learning was assessed using a modified version of the explicit sequential finger-tapping task introduced by Karni *et al*.^1^ In each experiment, participants were asked to execute either one of two equally difficult five-element finger-tapping sequences (Seq1: 4-1-3-2-4, Seq2: 1-4-2-3-1, where 1 = index finger, 2 = middle finger, 3 = ring finger, 4 = little finger) on a four-button keyboard with the right hand. Before the onset of the actual training session, participants had to correctly reproduce the assigned sequence three times in a row to verify explicit knowledge. The initial training session and the LateRT comprised 14 blocks of successive sequence execution, while the EarlyRT consisted of only 4 blocks. A green visual cue displayed on a computer monitor in front of the participants indicated the onset of a training block, while a stop signal (red visual cue) indicated the onset of a 25 s rest period. Participants were instructed to perform the finger-tapping sequence as fast as possible, while making as few errors as possible. Unbeknownst to the participants, each block of task execution was terminated after 60 button presses, which results in a maximum number of 12 correctly performed sequences per block. This design ensures that all participants perform the same number of finger movements regardless of task performance speed. Participants were instructed to refrain from mentally or physically practising the task in the consolidation period between the early and LateRT and to go about their regular daily business. During the ongoing study, we additionally introduced a visual analogue scale (range, 0–10) to assess subjective fatigue of the right hand before EarlyRT in the DBS-OFF and DBS-ON experiments (*n* = 11 patients).

### Data acquisition and statistical analysis

Custom MATLAB® (Mathworks, Natick, USA) scripts were used to record the timing of button presses on a customized gaming keyboard and to extract measures of speed performance and accuracy. Performance in terms of speed was defined as the average time (seconds) it took to execute a correct sequence within a given block of task execution (correct sequence duration, CSD). Accuracy was defined as the ratio of the number of correctly performed sequences per block to the maximum number of sequences per block (i.e. 12). As a primary measure of task performance, we applied a performance index (PI) that incorporates both equally important components of task performance as both speed and accuracy were modulated by DBS (see Results) and to take into account potential interindividual differences with respect to the strategy to improve task performance (e.g. focus on speed over accuracy, or vice versa):^22,34,42^


(1) $$\mathrm{PI}(x)=100\times e^{-\mathrm{CSD}(x)}\times e^{\mathrm{ACC}(x)-1}$$

where *x* is the block no., CSD(*x*)is the mean correct sequence duration in block *x*, and ACC(*x*) is the number of correctly performed sequences divided by 12 (i.e. the number of sequences per block) in block *x*.

Changes in task performance (PI/speed/accuracy) across blocks of practice and changes in the MDS-UPDRS-III/rUES values across time were explored by repeated measures ANOVAs. Follow-up rmANOVAs and paired-sample or independent-sample two-sided *t*-tests (as applicable) were used for a follow-up analysis in case rmANOVA revealed a significant interaction of within- or between-subject factors. Learning across initial training was assessed as the difference between the end-of-training performance (EOT, average PI across the last four blocks of the training session) and baseline task performance in the first block of the training session (beginning-of-training performance, BOT). Offline performance changes (i.e. consolidation) between the EarlyRT and LateRT were assessed as the difference between task performance at the beginning of the LateRT (PI of the first block) and the average performance across the four blocks of EarlyRT. We chose to use only the first block of LateRT for calculating offline performance changes to avoid confounding the consolidation measure with additional online learning. Associations of Parkinsonian motor symptoms and task performance gains were assessed with Pearson’s correlation coefficient (or Spearman’s rho in case the data were not normally distributed). All statistical analyses were performed with SPSS® 27 (IBM, Armonk, NY, USA) and MATLAB (Mathworks, Natick, USA). For all statistical tests, the alpha level was set to *P<* 0.05. rmANOVAs were checked for violation of sphericity and degrees of freedom, and *P*-values were corrected accordingly (Greenhouse-Geisser correction). For handling of missing data, see Supplementary Information 1.

### Electrode reconstruction, volumes of tissue activated and resting-state connectivity analysis

Volumes of tissue activated by DBS (VTAs) were estimated from individual preoperative MRI scans and postoperative computed tomography scans using 3DSlicer (v4.11.20210226, slicer.org^43^), the Lead-DBS toolbox (v2.5.3^44^), and SPM12 (v7487, Wellcome Trust Centre for Neuroimaging, London). Plausibility of the electrode reconstruction procedure was validated by a sweet-spot analysis using the VTAs (left DBS lead) and the DBS-associated differences of task-relevant motor symptoms (rUES scores). We then used publicly available resting-state functional MRI data from healthy controls included in the human connectome project to estimate VTA-to-whole-brain connectivity. In addition, *R*-maps were calculated as the voxel-wise correlation of VTA-to-voxel-connectivity with DBS-associated differences in performance gains during training and DBS-induced improvement of Parkinsonian motor symptoms to identify DBS-connectivity profiles that are associated with these metrics (for details, see Supplementary Information 2 and 3).

## Results

Demographic and clinical characteristics of PD patients and HCs included in the final analysis are provided in Table 1. Two PD patients had to be excluded. One did not tolerate DBS being turned off and terminated the experiment. The other did not manage to produce correct sequences when performing the motor learning task. As expected, PD patients and HCs differed significantly in terms of expression of Parkinsonian motor symptoms (baseline MDS-UPDRS-III DBS-ON experiment: 22.7 ± 8.4; HCs, 0.9 ± 1.3; *P<* 0.001). Additionally, PD patients differed significantly from HCs in terms of depressive symptoms (mean BDI DBS-ON experiment: 7.1 ± 4.6; HCs, 2.4 ± 3.0; *P* = 0.001), and there was a trend for lower MoCA scores in PD patients (MoCA DBS-ON experiment: 26.5 ± 2.9; range, 21–30; HCs, 28.4 ± 1.4; range of 26–30; *P* = 0.050). However, for both metrics, scores did not reach the threshold that would indicate mild depression or manifest dementia in either group.

**Table 1:** Demographic details of PD patients and healthy controls

Healthy controls			PD patients with subthalamic DBS									
ID	Sex	Age	ID	Sex	Age	DD	TI	DS	U ON	U OFF	LEDD	DBS parameters
1	M	61	1	M	58	10	57	R	16	32	1245	R (5,6,7)-, 1.0 mA, 50 µs, 130 Hz L (2,3,4)-, (5,6,7)-, 1.45 mA, 50 µs, 130 Hz
2	M	51	2	M	55	10	77	R	24	30	1968	R (5,6,7)-, 2.2 mA, 62 µs, 130 Hz L (2,3,4)-, 1 mA, 50 µs, 130 Hz
3	M	71	3	M	64	11	17	L	17	34	250	R (2,3,4)-, (5,6,7)-, 1.7 mA, 60 µs, 160 Hz L (2,3,4)-, 3.5 mA, 60 µs, 160 Hz
4	M	53	4	M	51	9	38	R	27	31	850	R 8-, 2.9 mA, 60 µs, 130 Hz L 8-, 2.7 mA, 60 µs, 130 Hz
5	F	53	5	M	55	12	97	L	29	39	200	R (5,6,7)-, 1.8 mA, 60 µs, 130 Hz L (5,6,7)-, 1.8 mA, 60 µs, 130 Hz
6	F	66	6	M	55	8	17	L	17	25	480	R (2,3,4)-, 4.0 mA, 60 µs, 130 Hz L (2,3,4)-, 2.7 mA, 60 µs, 130 Hz
7	F	56	7	M	52	8	11	R	16	39	240	R 3-,6-, 3.5 mA, 60 µs, 130 Hz L 5-, 2.4 mA, 60 µs, 130 Hz
8	M	65	8	F	58	15	27	L	40	61	850	R 2-,3-,4-, 3.75 mA, 60 µs, 130 Hz L 6-, 2.7 mA, 60 µs, 130 Hz
9	M	66	9	M	59	15	19	L	16	20	493	R 8-, 2.5 mA, 60 µs, 130 Hz L (5,6,7)-, 2.5 mA, 60 µs, 130 Hz
10	M	68	10	M	63	8	26	L	41	60	550	R 5-,7-, 3.5 mA, 60 µs, 130 Hz L 5-,6-, 4,2 mA, 60 µs, 130 Hz
11	F	56	11	M	58	7	14	R	38	46	300	R 5-,6-,7-, 2.8 mA, 60 µs, 130 Hz L 5-,6-,7-, 3.0 mA, 60 µs, 130 Hz
12	M	54	12	F	65	17	30	R	32	43	713	R 5-,6-,7-, 2.5 mA, 60 µs, 130 Hz L 5-,6-,7-, 2.5 mA, 60 µs, 130 Hz
13	M	64	13	F	49	5	44	R	27	53	550	R 8-, 2.35 mA, 50 µs, 130 Hz L 5-,6-,7-,8-, 3.0 mA, 50µs, 130 Hz
14	F	53	14	M	64	12	22	L	22	48	150	R 3-,4-, 3.1 mA, 60 µs, 130 Hz L 5-,7-, 2.0 mA, 60 µs, 130 Hz
15	M	50	15	F	68	9	4	R	13	59	200	R 5-,7-, 1.8 mA, 60 µs, 130 Hz L 5-,6-,7- 2.6 mA, 60 µs, 130 Hz
16	M	67	16	F	35	5	18	R	28	63	900	R 5-,6-,7-, 2.4 mA, 60 µs, 130 Hz L 5-,6-,7-, 2.2 mA, 60 µs, 130 Hz
17	F	63	17	M	56	20	59	L	13	42	240	R 1-, 3.2 mA, 60 µs, 180 Hz L (5,6,7)-, 2.8 mA, 60 µs, 180 Hz
18	M	55	18	F	65	21	4	R	18	35	600	R 2-,3-,4-, 1.5 mA, 60 µs, 130 Hz L 5-,6-,7-, 2.5 mA, 60 µs, 130 Hz
19	M	56	19	F	64	15	12	L	20	36	500	R 5-,6-,7-, 2.8 mA, 60 µs, 130 Hz L 2-,3-,4-, 2.8 mA, 60 µs, 130 Hz
		59.4 ± 6.6			57.6 ± 7.7	11.4 ± 4.6	31.2 ± 25		23.9 ± 9	41.9 ± 13	594 ± 444	

The designation of DBS electrode contacts is defined as follows (standardized for different manufacturers and device types): 1 and 8 denote the most caudal and cranial contacts, respectively; 2, 3, 4 and 5, 6, 7 denote the individual segments of the middle caudal and cranial contacts of directional electrodes. For non-directional electrodes, the middle caudal and cranial contacts are designated as (2,3,4)− and (5,6,7). Mean ± standard deviation provided in the two bottom rows. Abbreviations: M = male; F = female; DD = disease duration (years); TI = time interval since DBS implantation (months); DS = dominant side of PD motor symptoms (L = left; R = right); LEDD = levodopa equivalent dose (mg); U DBS-ON = pre-Training MDS-UPDRS III assessment under DBS-ON; U DBS-OFF = pre-Training MDS-UPDRS III during inactive DBS; *R* = stimulation of the right subthalamic nucleus; *L* = stimulation of the left subthalamic nucleus.

### DBS effects on Parkinsonian motor symptoms

**Figure 2:**
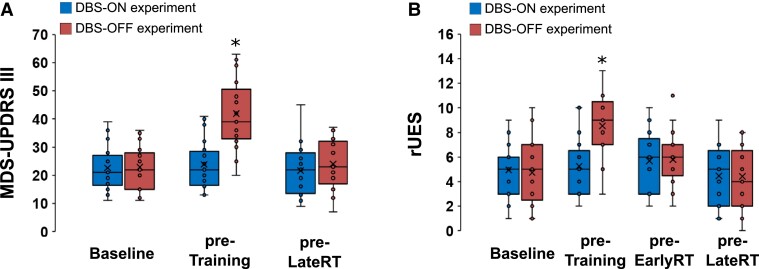
**Effects of subthalamic DBS on global and specifically task-relevant Parkinsonian motor symptoms.** (**A**) Boxplots display MDS-UPDRS-III scores of PD patients for the DBS-ON and DBS-OFF experiments across Baseline, immediately before Training (pre-Training), and before late retesting (pre-LateRT). (**B**) Right upper extremity MDS-UPDRS-III subscores (rUES) of PD patients for the DBS-ON and DBS-OFF experiments across Baseline, immediately before Training (pre-Training), immediately before early retesting (pre-EarlyRT), and before late retesting (pre-LateRT). Asterisk indicates statistically significant difference for all within-experiment (across time) and between-experiments (DBS-ON/DBS-OFF) comparisons (*P* < 0.05, *post hoc* paired *t*-tests, *n* = 19).

Baseline MDS-UPDRS-III averaged 22.7 ± 8.4 in the DBS-ON experiment and 22.9 ± 8.1 in the DBS-OFF experiment (*P* = 0.831) demonstrating a similar baseline burden of PD motor symptoms at the beginning of both experiments. A rmANOVA conducted on the MDS-UPDRS-III scores with the main factors DBS (DBS-ON/DBS-OFF) and Time (Baseline/pre-Training) revealed a significant interaction of DBS × Time [*F*(2,36) = 40.604, *P* < 0.001]. This was driven by almost doubled MDS-UPDRS-III scores in the pre-Training assessment compared with baseline in the DBS-OFF experiment (41.9 ± 12.7), while motor symptom severity remained unchanged in the DBS-ON experiment (23.9 ± 9.0; pre-Training MDS-UPDRS-III scores DBS-ON versus DBS-OFF, *P<* 0.001, Fig. 2A). To specifically explore those Parkinsonian motor symptoms that more directly affected performance in the motor learning task, we also applied a rmANOVA with the main factors DBS and Time to the rUES values, which revealed a highly significant interaction of both factors [*F*(3,54) = 15.041, *P* < 0.001]. This interaction was driven by the observation that turning DBS off led to increased (i.e. worse) rUES values in the pre-Training assessment (8.5 ± 2.7) that differed significantly from the pre-Training rUES values under active DBS (5.3 ± 2.4, *P<* 0.001), whereas the rUES did not significantly differ at any other time point (baseline, pre-EarlyRT, pre-LateRT; all ≥0.480; Fig. 2B). Collectively, this demonstrates that DBS had a significant and clinically relevant effect on Parkinsonian motor symptoms. Of note, post-Training right-hand fatigue was significantly higher in the DBS-OFF experiment compared with the DBS-ON experiment despite ‘normalized’ rUES values (mean visual analogue scale value, DBS-ON: 3.6 ± 2.0, DBS-OFF: 5.9 ± 2.7, *P* = 0.029, *n* = 11).

### DBS modulates both speed and accuracy of task performance

**Figure 3:**
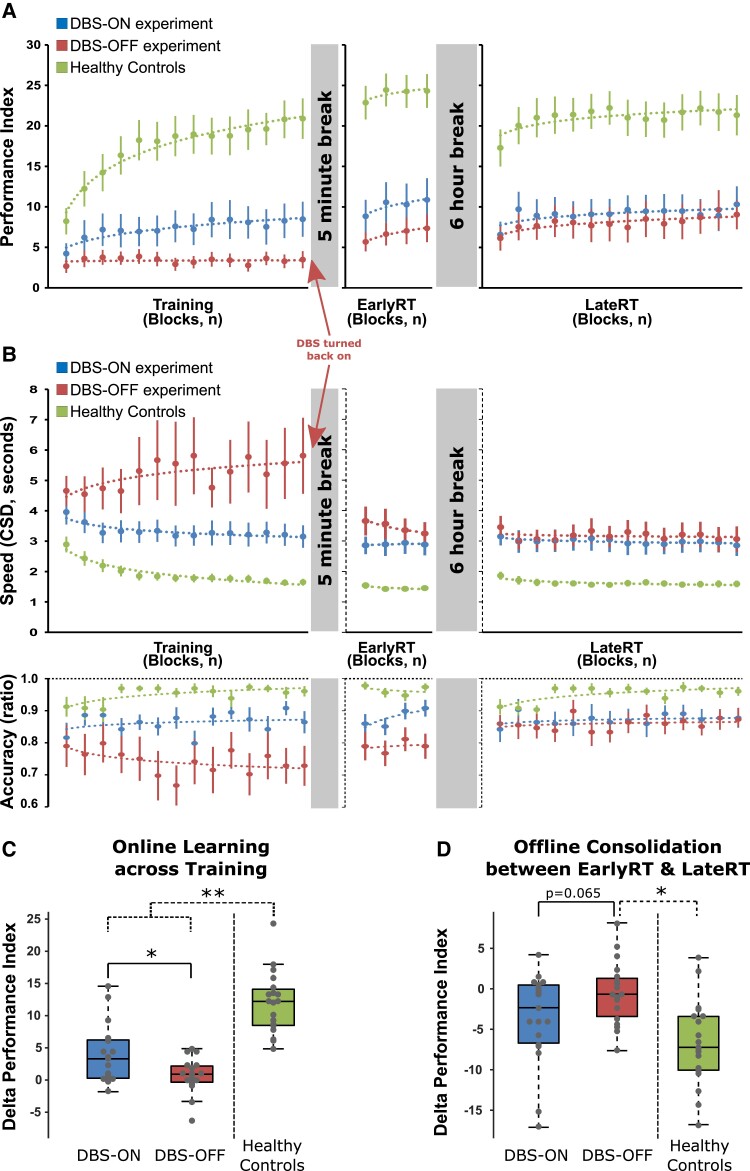
**Motor sequence-learning task performance.** (**A**) PI values for healthy controls (*n* = 19) and PD patients (*n* = 19) during the DBS-ON and DBS-OFF experiments across blocks of the training session (Training), early retesting (EarlyRT) and late retesting (LateRT). Please note that in the DBS-OFF experiment DBS was only turned off during the initial training session. (**B**) Task performance in terms of speed (average duration to perform a correct sequence per block, CSD) and accuracy (proportion of correctly performed sequences per block) across blocks of Training, EarlyRT and LateRT. Error bars indicate the standard error of the mean; dotted lines represent the ‘denoised’ logarithmic trendline across blocks of practice in (**A**) and (**B**). (**C**) Boxplots display the magnitude of online learning across the initial training session (difference between the mean PI across the last four blocks at the end of the training session and initial training performance in the first block of Training) in healthy controls and PD patients under DBS-ON and DBS-OFF. Within the PD patient group, DBS-ON and DBS-OFF conditions were compared using a paired *t*-test indicating that training-induced performance improvements across the initial training session differed significantly between DBS conditions [*t(18) = 2.519, *P* = 0.021]. Exploratory between-group comparisons of training-induced performance improvements across initial training of healthy controls with PD patients’ performance under DBS-ON and DBS-OFF experiments were performed by applying separate (between group, dotted brackets) unpaired *t*-tests [healthy controls versus PD patients DBS-ON, ***t(36) = −5.379, *P* < 0.001; healthy controls versus PD patients DBS-OFF, ***t(36) = −8.967, *P* < 0.001; Bonferroni corrected]. (**D**) Boxplots display the magnitude of offline performance changes within the 6 h between early and late retesting (i.e. consolidation, difference between the PI in the first block of late retesting and the mean PI across the four blocks of early retesting) in healthy controls and PD patients during the DBS-ON and DBS-OFF experiments. Similar to the comparison of the initial online learning performance, a paired *t*-test was applied to compare consolidation between the DBS-ON and DBS-OFF experiments within the PD patient group [*t*(18) = −1.963, *P* = 0.065]. Two separate unpaired *t*-tests were applied for between-group comparisons (dotted brackets) of consolidation in healthy controls and in PD patients in the DBS-ON and DBS-OFF experiments [healthy controls versus PD patients DBS-OFF, ***t(36) = 4.137, *P* < 0.001, Bonferroni corrected; healthy controls versus PD patients DBS-ON, *t*(36) = 1.788, *P* = 0.082].

RmANOVA applied to the speed (CSD) measures across Blocks of the initial training session revealed a significant main effect of DBS [*F*(1,18) = 6.184, *P* = 0.023] that was driven by faster sequence execution in the DBS-ON condition (mean CSD across Training, 3.3 ± 1.6 s) compared with the DBS-OFF condition (5.2 ± 4.2 s). Accuracy was modulated by DBS in a similar direction as mean accuracy across the initial training session amounted to 0.86 ± 0.14 in the DBS-ON experiment and 0.74 ± 0.28 in the DBS-OFF experiment [DBS, *F*(1,18) = 4.426, *P* = 0.050; Fig. 3B]. As both equally important components of the motor learning task were modulated by DBS, and in order to account for potential inter-individual differences with respect to the strategy to improve task performance (prioritize speed over accuracy or vice versa), all further analyses were performed based on a PI that incorporates both speed and accuracy.

### Suspending DBS prevents performance gains during training

A rmANOVA conducted on the PI values across blocks of training with the within-subject factors DBS and Block revealed a significant main effect of DBS [*F*(1,18) = 7.070, *P* = 0.016], a significant main effect of Block [*F*(4.66,83.96) = 4.460, *P* = 0.002], and a significant interaction of DBS × Block [*F*(4.32,77.79) = 3.309, *P* = 0.013]. As expected, the significant main effect of DBS was driven by impaired overall task performance across blocks of training in the DBS-OFF condition (average PI across training session, 3.37 ± 3.48) compared with the DBS-ON condition (7.33 ± 8.62, Fig. 3A). Follow-up rmANOVAS to explore the significant interaction of DBS × Block demonstrated a highly significant effect of Block in the DBS-ON condition [*F*(3.92,70.52) = 5.733, *P<* 0.001], while there was no significant change of task performance across the training session (i.e. no behavioural evidence of learning) in the DBS-OFF condition [*F*(4.27,8.18) = 0.973, *P* = 0.431]. The significant effect of Block in the DBS-ON condition was driven by an average individual PI improvement across training of 3.87 ± 4.53 (difference of PI at EOT minus BOT), while PI improved by only 0.62 ± 2.79 during training in the DBS-OFF condition (*P* = 0.021, Fig. 3C).

### DBS partly restores training-induced performance gains

Training performance in the DBS-ON and DBS-OFF experiments was compared with age-matched HCs by applying mixed rmANOVAs to the PI values with the between-subject factor Group (PD patients, HCs) and the within-subject factor Block separately for DBS-ON and DBS-OFF. In addition to a significant main effect of Block [*F*(6.54,235.42) = 31.908, *P* < 0.001], a significant main effect of Group [*F*(1,36) = 11.426, *P* = 0.002] was found, driven by significantly higher PI values averaged across training in HCs (17.39 ± 9.69) compared with PD patients under active DBS (7.33 ± 8.62). More importantly, different dynamics of performance gains in DBS-ON and HCs were indicated by a significant interaction of Group × Block [*F*(6.54,235.42) = 10.119, *P* < 0.001, Fig. 3A]. This interaction was driven by larger PI gains in HCs than in PD patients under DBS-ON (PI EOT minus BOT, HCs: 11.99 ± 4.77, PD patients DBS-ON: 3.87 ± 4.53, Fig. 3C). As expected, the comparison of HCs’ and PD patients’ DBS-OFF performance revealed a highly significant main effect of Group [*F*(1,36) = 35.204, *P* < 0.001] and interaction of Group × Block [*F*(6.53,234.98) = 21.211, *P<* 0.001] generated by the overall low task performance and lack of performance improvements across training in PD patients under inactive DBS compared with HCs. Collectively, the above findings indicate that suspending subthalamic DBS inhibits the ability of PD patients to improve task performance across the training session. Active DBS, however, restored the motor learning abilities of PD patients leading to increased training-induced performance gains, yet not up to the level of age-matched HCs.

### Early retest

RmANOVA conducted on the four blocks of EarlyRT revealed a significant main effect of DBS [*F*(1,16) = 5.858, *P* = 0.026] that was driven by impaired task performance in the DBS-OFF experiment compared with the DBS-ON experiment (mean PI across EarlyRT, DBS-OFF: 6.68 ± 6.28, DBS-ON: 10.14 ± 10.50). There was a significant effect of Block [*F*(1.88,33.89) = 3.694, *P* = 0.038], but no significant interaction of DBS × Block [*F*(3,54) = 0.304, *P* = 0.822], indicating similar learning dynamics during EarlyRT (Fig. 3A). Comparison of PD patients’ DBS-ON and DBS-OFF average EarlyRT PI with HCs revealed significantly better performance of HCs (23.97 ± 8.76) than PD patients DBS-ON and DBS-OFF (both *P<* 0.001), but no significant interaction of Group × Block in both cases (both *P* ≥ 0.860). Collectively, this demonstrates that after DBS was turned back on in the DBS-OFF experiment, the capacity to learn across EarlyRT did not differ from that in the DBS-ON experiment and from that of HCs.

### Late retest inhibition of initial performance gains under inactive DBS does not compromise consolidation

RmANOVA on PI values across blocks of the LateRT session revealed a significant effect of Block [*F*(4.97,89.36) = 3.110, *P* = 0.013], but no significant effect of the DBS condition during Training [*F*(1,18) = 2.060, *P* = 0.168], and no significant interaction of both factors [*F*(5.33,96.00) = 1.105, *P* = 0.364], indicating a similar level of overall task performance and similar learning across LateRT in the DBS-OFF and DBS-ON experiments (Fig. 3A). Average LateRT PI in PD patients was significantly lower than in HCs in both the DBS-ON and DBS-OFF experiments (both *P* < 0.001).

Consolidation across the 6 h offline interval was explored by applying a rmANOVA with the factors DBS and Session (EarlyRT/LateRT) to the average PI across the four blocks of EarlyRT and the PI at the onset of LateRT (first block). In addition to a significant effect of DBS [*F*(1,18) = 6.902, *P* = 0.017] and a significant effect of Session [*F*(1,18) = 7.034, *P* = 0.016], there was a trend for a significant interaction of DBS × Session [*F*(1,18) = 3.854, *P* = 0.065], suggesting that offline performance gains differed between the DBS-ON and DBS-OFF experiments. This was driven by the fact that performance decreased from Early RT (mean PI across EarlyRT, 10.14 ± 10.50) to the first block of LateRT (PI, 6.57 ± 7.00, *P* = 0.012) in the DBS-ON experiment, while task performance remained stable in the DBS-OFF experiment (mean PI across EarlyRT: 6.68 ± 6.28, PI first block of LateRT: 6.13 ± 6.68, *P* = 0.542). HCs showed a similar pattern of consolidation as PD patients in the DBS-ON experiment in that task performance decreased significantly offline (mean PI across EarlyRT: 23.97 ± 8.76, PI first block of LateRT: 17.28 ± 10.00, *P<* 0.001). Accordingly, comparison of consolidation between HCs and PD patients in the DBS-ON and DBS-OFF experiments by separate rmANOVAs revealed a significant main effect of Group in both cases (both *P<* 0.001), while a significant interaction of Group × Session was only found for the comparison of HCs and DBS-OFF (*P<* 0.001; HCs versus DBS-ON, *P* = 0.082, Fig. 3D). Moreover, we found a highly significant negative correlation of individual DBS-associated differences in performance gains during training with DBS-associated differences in offline consolidation (rho = −0.602, *P* = 0.006). This supports that inactive DBS-associated inhibition of performance gains across training was later compensated during offline consolidation.

### Correlations of DBS-induced effects on learning with effects on Parkinsonian motor symptoms

**Figure 4:**
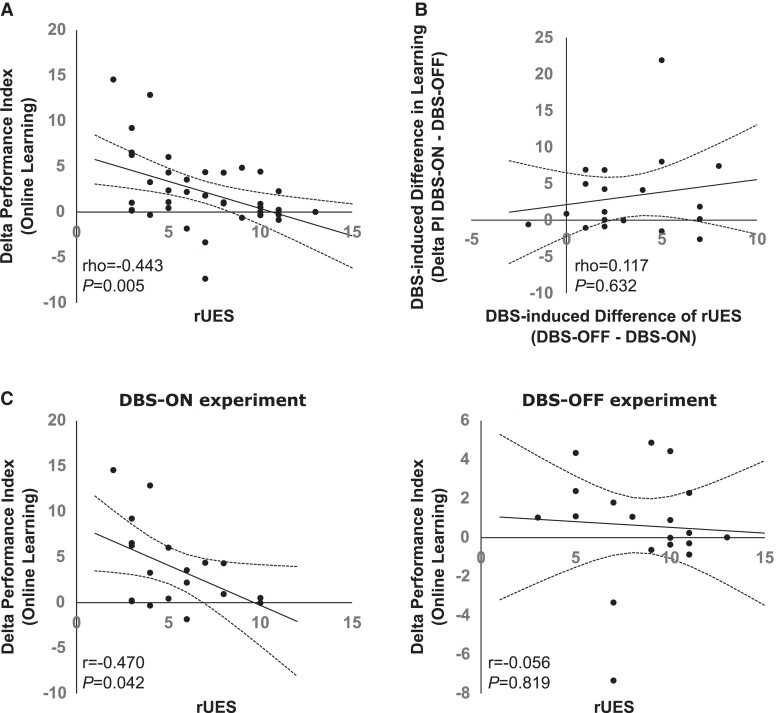
**Association of training-induced performance gains and Parkinsonian motor symptoms.** (**A**) Correlation of the right upper extremity subscore (rUES, bradykinesia and rigidity items) of the MDS-UPDRS-III with performance gains during initial training (‘online learning’, delta average PI values across the last four blocks of the training session minus PI values in the first block of training) across the DBS-ON and DBS-OFF experiments showing a significant negative association of Parkinsonian motor symptom expression and performance gains during the training session (Spearman’s rho). (**B**) Correlation of the individual DBS-induced improvement of Parkinsonian motor symptoms (delta between pre-Training rUES in the DBS-OFF versus the DBS-ON experiment) and DBS-associated differences of performance gains during training (delta performance gains DBS-ON minus performance gains DBS-OFF) indicating no significant association of DBS-associated changes of performance gains during training and DBS-associated improvements in Parkinsonian motor symptoms (Spearman’s rho). (**C**) Significant negative correlation (Pearson correlation coefficient) of Parkinsonian motor symptom expression (rUES) and online learning during training in the DBS-ON experiment (left panel), while Parkinsonian motor symptom expression was not significantly associated with online learning during training in the DBS-OFF experiment (right panel). Scatter plots, solid line displays the best linear fit, and dotted lines show the lower and upper 95% confidence intervals.

Across both experiments (DBS-ON and DBS-OFF), the magnitude of performance gains during training was negatively associated with pre-Training task-relevant Parkinsonian motor symptoms (rho = −0.443, *P* = 0.005, *n* = 38), indicating that a worse motor state was associated with impaired performance gains during training (Fig. 4A). However, no significant associations were found between the magnitude of the individual DBS-induced improvements of motor symptoms (delta pre-Training rUES in the DBS-OFF versus the DBS-ON experiment) and the DBS-induced differences of performance gains during training (rho = 0.117, *P* = 0.632, *n* = 19, Fig. 4B). Separate correlation analyses of the association of the PD motor symptoms as assessed by rUES and PI gains across initial training in the DBS-ON experiment and the DBS-OFF experiments demonstrated that, while PD motor symptom expression significantly correlated with performance gains in the DBS-ON experiment (*R* = −0.470, *P* = 0.042, *n* = 19), this correlation was lost when DBS was turned off (*R* = −0.056, *P* = 0.819, *n* = 19, Fig. 4C).

### DBS sweet-spot and resting-state connectivity analysis

**Figure 5:**
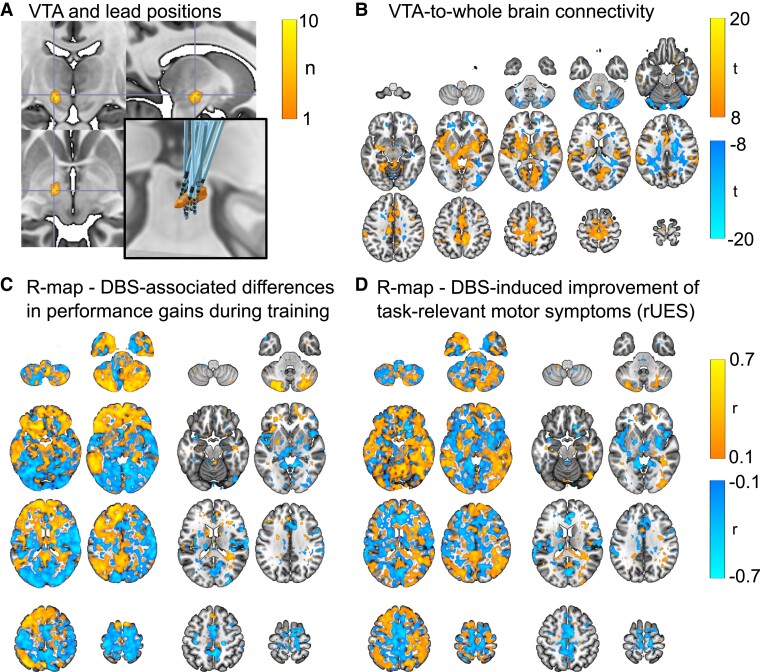
**Volumes of tissue activated by DBS, DBS sweet-spot and *R*-maps.** (**A**) Overlay of simulated volumes of tissue activated by DBS (VTA, left lead, *n* = 19 PD patients). The brighter the colour, the more individual VTAs were reaching the corresponding voxel. The centre of the cross indicates the calculated stimulation sweet-spot for optimal improvement of PD motor symptoms. The panel on the bottom right shows the left DBS lead locations relative to the left STN (view from the front). (**B**) Voxels that showed significant normative resting-state connectivity to the VTAs [*t*-tests at voxel-level, *P*(FWE) < 0.05]. (**C** and **D**) *R*-maps calculated as the voxel-wise correlation of VTA-to-voxel-connectivity with the DBS-associated differences in performance gains during training (**C**), and DBS-induced improvement of Parkinsonian motor symptoms (**D**). The complete non-thresholded *R*-map is depicted on the left of each panel, while on the right the maps are masked using the map of significant VTA-to-whole-brain connectivity voxels (**B**).

We found our DBS sweet-spot to improve task-relevant Parkinsonian motor symptoms to be at MNI coordinates (−12.3, −13.7, −5.7 mm) which correspond well with previously published coordinates.^45^ The mean distance of the VTA centres of mass to the sweet-spot coordinate was 2.7 ± 1.1 mm (Fig. 5A). This internal quality validation indicated plausible electrode reconstruction results and stimulation sites.

In the normative resting-state functional MRI analysis, we found significant positive VTA-to-whole-brain connectivity to bilateral basal ganglia, insular, frontal, perisylvic and precentral regions, while negative connectivity was found to cerebellar and occipital areas (Fig. 5B). We further analysed whether connectivity between individual VTAs and particular clusters correlated either with DBS-associated differences in performance gains during training or with DBS-induced modulation of task-relevant Parkinsonian motor symptoms. However, we found no evidence for such associations (Family-wise Error-corrected at cluster level, DBS-induced differences of performance gains *P ≥* 0.682, DBS-induced motor symptom improvement *P ≥* 0.997 for every cluster). We then calculated *R*-maps to further explore VTA-connectivity associated with DBS-related differences in performance gains and DBS-induced improvement of Parkinsonian motor symptoms. The unthresholded *R*-maps exhibited both positive and negative correlations, albeit in partly different locations. DBS-associated differences in performance gains during training had positive correlations to VTA-connectivity to cerebellar, frontal and parietotemporal regions particularly in the left brain, while negative correlations were found with the central region. DBS-induced improvement of task-relevant motor symptoms was correlated with positive VTA-connectivity to bilateral parietotemporal and frontal areas, while negative connectivity to central regions was also correlated with improvements in rUES. The pattern was retained, although attenuated, when masking the *R*-maps with the map of voxels whose connectivity to the VTAs was significant (Fig. 5C and D). The similarity of the *R*-maps for the DBS-associated differences in performance gains during training and for DBS-induced improvements of motor symptoms was low (*R* = 0.201, *P* = 0.208). Following the approach by de Almeida Marcelino *et al*.,^28^ the correlation between the behavioural metrics and the individual similarities of connectivity maps to the *R*-map for the same metric was significant for DBS-associated differences in performance gains during training (*R* = 0.721, *P<* 0.001) and DBS-associated motor symptom improvement (*R* = 0.643, *P* = 0.003). However, as *R*-maps are built upon the metric with which they are correlated, this result arises directly from the way *R*-maps are constructed and, therefore, does not generate new insights. We, additionally, used leave-one-out cross-validation to predict DBS-induced differences in performance gains during training and DBS-induced motor symptom improvement from the similarity of individual connectivity maps to the *R*-maps generated by leaving out the patient whose behavioural metric was to be predicted. We found that neither DBS-associated performance gains during training nor DBS-induced improvements in Parkinsonian motor symptoms could be predicted using this approach (*R*^2^ ≤ 0.134, *P* ≥ 0.123).

## Discussion

The current study investigated the effects of subthalamic DBS on motor sequence learning during initial training and subsequent offline consolidation in PD. We found that task performance gains were inhibited during initial training with inactive DBS as expected. While performance improved significantly during training under active DBS, patients with PD did not reach the level of task performance and learning dynamics of age-matched HCs. Importantly, motor sequence performance after an offline consolidation interval of 6 h was similar in PD patients regardless of whether the initial training session had been performed under active or inactive DBS. This could indicate that despite severe impairments of training-induced improvements in motor sequence execution under inactive DBS, formation of motor sequence memory traces to be later consolidated offline may still be intact. Interestingly, inhibition of performance gains during initial training in PD patients under inactive DBS was compensated during offline motor memory consolidation, while motor performance decreased across the offline consolidation interval in both age-matched HCs and PD patients when initial training was performed under active DBS.

Motor learning is traditionally defined as an improvement in motor performance through practice. According to this definition and if only the initial training session of our study is considered, our results are in line with previous evidence, suggesting that subthalamic DBS improves early motor learning compared with impaired learning under inactive DBS.^28,29^ However, withdrawal of DBS, by design, results in clinically relevant worsening of Parkinsonian motor symptoms, which contaminates the behavioural readout of motor learning by limiting overall motor performance and, consequently, the range of training-induced performance gains. It is impossible to distinguish the effects of DBS on motor learning from temporary DBS-associated effects on motor performance in this context. However, this distinction is essential as conditions that affect execution do not necessarily have to affect learning.^37^ The alternative interpretation for the assumption that DBS improves early motor learning is that impaired motor execution due to DBS withdrawal prevented any training-induced performance improvements that could have indicated online learning. Different levels of contamination of performance gains with impairments of motor execution (e.g. due to differences in dopaminergic medication or disease stage) may also at least partly explain the inconsistent results of previous research in terms of whether motor sequence learning is impaired in PD or not.^16-18,20,22,23,46^ Our current results suggest that not only general motor execution but also learning dynamics during initial training were still significantly impaired in PD compared with age-matched healthy controls despite ‘best possible’ treatment with subthalamic DBS and dopaminergic medication. However, PD patients on active DBS continued to show clinically relevant impairments of motor execution due to remaining Parkinsonian motor symptoms. Therefore, we cannot rule out the possibility that the apparent differences between PD patients and HCs in terms of early learning dynamics in the current study are still actually driven by differences in motor execution.

Importantly, the ultimate goal of motor training is not to temporarily promote motor performance during the period of practising, but to induce sustained improvements in motor skills. Moreover, given that performance-learning distinction is especially evident under challenging training conditions (which is the case in a PD patient who has been withdrawn from DBS), delayed recall of training-induced learning after consolidation may represent a more valid, however indirect, indicator of how much was actually learned during training.^37^ A link between the neural substrates underlying, on the one hand, online encoding of motor sequence memory traces during training and, on the other hand, offline consolidation, is provided by several functional imaging studies which demonstrated that training-induced changes of motor task-related activity and connectivity predict subsequent consolidation.^32,34-36^ This could mean that activation in an online motor learning-related network during training is necessary to induce consolidation. However, in the current study, motor sequence performance after the 6 h offline consolidation interval did not relevantly differ regardless of whether initial training was performed under ‘optimal’ (active DBS) or impaired (inactive DBS) motor execution conditions. This may indicate that encoding of motor sequence memory traces during initial training was similarly effective under active and inactive DBS despite severely impaired task execution under the latter condition. One could still argue that the significant difference with respect to sequence performance during the EarlyRT may well indicate improved early learning under active DBS since worsened motor symptoms under inactive DBS had already recovered to the level of the DBS-ON experiment at this time. However, PD patients reported significantly higher hand fatigue after training under inactive DBS despite ‘normalization’ of task-relevant Parkinsonian motor symptoms. As neuromuscular fatigue itself may degrade task execution,^47,48^ this might at least partly explain the performance differences across early retesting without having to assume relevant differences in prior learning. However, even if task performance differences during early retesting were indeed indicative of improved learning under active DBS, this advantage is lost across the offline consolidation interval. A similar pattern was observed in age-matched HCs indicating that the ability of PD patients to consolidate training-induced performance gains is not inferior to that of age-matched HCs. This corroborates previous evidence showing similar overnight and over-the-day motor memory consolidation in PD and age-matched HCs.^22,24,25^ The observation that consolidation was even relatively enhanced in PD patients following prior training under inactive DBS further supports that the capacity to consolidate motor memories is relatively intact in PD. Importantly, we do not suggest that suspending DBS during training enhances later motor memory consolidation. Rather, intact offline motor memory consolidation in PD was the precondition to compensate for the inhibition of performance gains during initial training when the ability to improve performance—but not the ability to learn—was taken by the withdrawal of DBS.

We then explored whether specific DBS-connectivity patterns could be identified that underlie DBS-associated effects on early motor learning. In line with previous research, the resting-state fMRI analysis demonstrated plausible significant connectivity between the VTAs and several cortical areas in a cohort of healthy participants. Significant connectivity between subthalamic nucleus (STN) and the primary motor cortex is in line with studies demonstrating the hyperdirect pathway,^49^ direct alteration of M1 excitability by DBS^50^ and reduction of M1 activity by DBS over time.^51^ The significant connectivity to frontal perisylvic areas (e.g. SMA) and basal ganglia is plausible and clinically relevant.^49,52-54^ However, no significant voxels of VTA-to-whole-brain connectivity were associated with DBS-dependent differences in performance gains during training (‘early learning’) or Parkinsonian motor symptom improvements. Following the approach by de Almeida Marcelino *et al*.,^28^ we calculated *R*-maps for DBS-associated differences in performance gains during training and, in addition, DBS-induced motor symptom improvements. The resulting *R*-map pattern for DBS-induced motor symptom improvements with positive correlations to frontal and parietotemporal regions and negative correlations to central regions is in line with previous results.^55^ The *R*-map pattern for DBS-induced differences in performance gains during training was different from the pattern associated with DBS-induced improvements of motor symptoms and visually similar to the connectivity pattern that was associated with DBS-induced effects on ‘early learning’ in de Almeida Marcelino *et al*.^28^ Different connectivity patterns of DBS-associated differences in early learning and Parkinsonian motor symptom improvements may seem to support a specific role of subthalamic DBS in motor learning apart from facilitating motor execution in PD. However, since *R*-maps are created based on the behavioural metrics, the contamination of behavioural metrics by the learning-performance distinction problem also confounds the generation of the *R*-maps. Because the DBS-induced differences in performance gains during training and DBS-induced motor symptom improvements were not correlated at the behavioural level, it is not surprising that a lack of association was found between these parameters when expressed in terms of connectivity in the joint fMRI data set. Different *R*-map patterns of connectivity related to DBS-associated differences in ‘learning’ and improvements of Parkinsonian motor symptoms, thus, do not indicate specific subthalamic DBS involvement in inherent motor learning processes.

Our findings corroborate current concepts regarding the role of the STN in motor control. Both the striatum and the STN are input structures to the basal ganglia and are subdivided into sensorimotor, associative and limbic subareas based on cortical connectivity.^52,56-58^ However, their function in motor control and motor learning likely differs. Specifically, it has been shown that within the striatum, early motor learning is supported by the activation of associative subareas, whereas activation of sensorimotor subareas of the striatum is involved in the execution of already well-learned sequences.^11,59-61^ The sensorimotor STN, however, rather affects motor execution by modulating the excitatory control over the inhibitory basal ganglia motor output to the thalamus and the cortex.^49,56,57,62^ Facilitation of movement execution as mediated by the STN is associated with suppression of beta oscillations in the STN that is tightly linked to movement preparation and movement execution.^63-68^ In PD, pathologically exaggerated beta oscillations were linked to motor symptoms such as bradykinesia and rigidity.^66,69-71^ Although the exact mechanisms of how DBS improves Parkinsonian motor symptoms remain elusive, suppression of pathologically increased beta oscillations by high-frequency interruption of the sensorimotor STN is likely one key mechanism.^72,73^ However, given the above evidence that motor execution and motor learning rely on finely tuned temporal dynamics of beta oscillations in the STN and the associated cortico-basal ganglia circuits, it seems unlikely that constant unspecific disruption of STN activity by DBS may facilitate such complex and timing-critical processes in a specific manner. These considerations are in line with our current findings that indicate a fundamental role of the STN in global motor control that is well accessible to modulation by DBS, while modulation of STN activity by DBS had no relevant effect on motor learning itself when DBS-induced effects on motor execution were accounted for. However, DBS-associated changes in performance gains across initial training did not significantly correlate with DBS-induced modulation of Parkinsonian motor symptoms despite a generally strong negative association of performance gains during training and Parkinsonian motor symptom load. Like in previous studies, this may be interpreted as evidence that DBS modulates motor learning independent of effects on motor execution. However, the decorrelation of these metrics is, again, likely driven by the inhibition of training-induced performance gains in the DBS-OFF experiment due to severe impairments in motor execution (‘one cannot improve, while one cannot move’). Of note, when DBS was on (i.e. the motor state allowed performance improvements), training-induced performance gains were significantly correlated with PD motor symptom expression. This indirectly supports that DBS rather provides the precondition to improve performance by facilitating motor execution than to directly modulate motor learning.

## Conclusion

Although subthalamic DBS led to a well-established improvement in Parkinsonian motor symptoms and motor execution, the current study showed that subthalamic DBS had no specific effects on motor learning. This is evidenced by the fact that task performance at delayed retesting was similar across experiments despite large DBS-dependent differences in motor execution during initial learning. Moreover, despite plausible patterns of VTA-to-whole-brain connectivity, we found no particular connectivity patterns that suggested a role of the stimulated portion of the STN in motor learning when contrasted to the role of the STN in Parkinsonian motor symptom modulation. This supports that the STN has an important role in general motor control, while it does not modulate motor learning, at least not directly. Importantly, offline consolidation was intact in PD patients as inhibition of motor performance gains during initial training under inactive DBS was later compensated across offline consolidation. From a clinical perspective, this suggests that it is not necessary for PD patients to wait for an optimal motor state to practice a new motor skill, as longer-term outcomes, including an offline consolidation phase, appear to be independent of the magnitude of performance gains during initial training.

## Supplementary Material

fcad070_Supplementary_Data

## Data Availability

Data privacy statements signed by all subjects protect personal data. The data can be made available upon specific request taking into account the opinion of the local data privacy board.

## Abbreviations

BDI =: Beck’s Depression Inventory (short version)
BOT =: beginning-of-training performance
CSD =: correct sequence duration
DBS =: deep brain stimulation
DBS-ON =: deep brain stimulation turned on
DBS-OFF =: deep brain stimulation turned off
EarlyRT =: early retest
EOT =: end-of-training performance
HCs =: healthy controls
LateRT =: late retest
MDS-UPDRS-III =: Movement Disorder Society Unified Parkinson’s Disease Rating Scale
MNI =: Montreal Neurological Institute coordinate system
MoCA =: Montreal Cognitive Assessment
PD =: Parkinson’s disease
PI =: performance index
rmANOVA =: repeated measures ANOVA
rUES =: right upper extremity subscore of the MDS-UPDRS-III
STN =: subthalamic nucleus
VTA =: volume of tissue activated by deep brain stimulation
