# Supplementary material for: Motor learning is independent of effects of subthalamic deep brain stimulation on motor execution
Source: Brain Commun. 2023 Mar 17;5(2):fcad070. doi: 10.1093/braincomms/fcad070 (PMC10065184; doi:10.1093/braincomms/fcad070)
Supplement: fcad070_Supplementary_Data [file fcad070_supplementary_data.docx]

**Supplementary Material**

**Materials and Methods**

**Supplementary information 1: Analysis of behavioral data – missing data**

One patient with Parkinson’s disease (PD) discontinued the training session in the DBS-OFF experiment after eleven blocks due to severe hand fatigue. For this patient the missing task performance assessments in the last three blocks of the training session were each replaced by the performance in block eleven. In case no correct sequences were performed within a block of task practice (one training block each in two PD patients in the DBS-OFF experiment), PI of that block was set to zero and the missing corresponding speed performance values were imputed by the mean values of the previous and the following block. The same procedure was applied for one block of training during the DBS-OFF experiment, in which the speed performance (correct sequence duration, CSD) deviated strongly (by more than 2.5 standard deviations) from the mean DBS-OFF training performance of that participant.

**Supplementary information 2: Deep brain stimulation electrode reconstruction and estimation of volumes of tissue activated by deep brain stimulation**

Volumes of tissue activated by the left DBS lead (VTA) were estimated from individual preoperative MRI scans and postoperative CT scans using 3DSlicer (v4.11.20210226, slicer.org)^1^, the Lead-DBS toolbox (v2.5.3)^2^, and SPM12 (v7487, Wellcome Trust Centre for Neuroimaging, London). Following conversion to Nifti file format using 3DSlicer, postoperative CT scans were coregistered to corresponding individual preoperative MRI scans. In a first step, this procedure was performed manually and as precisely as possible using 3DSlicer, followed by an automatic coregistration step with a two-step linear registration (rigid and affine transformation) as implemented in Advanced Normalization Tools^3^ (http://stnava.github.io/ANTs/) via Lead-DBS. Coregistered scans were then spatially normalized into MNI space using the SyN registration approach implemented in Advanced Normalization Tools^4^. Nonlinear deformation into template space was implemented using two steps (rigid followed by affine transformation) followed by a nonlinear (whole brain) SyN registration stage. DBS-electrode trajectories were semi-automatically detected and approximated as implemented in Lead-DBS^2^. Precise electrode location was manually determined and refined. For Boston Scientific Active Tip segmented electrodes (*n*=7 PD patients) we used the algorithm by Dembek and colleagues^5^ as implemented in Lead-DBS to determine the electrode orientation, which was manually refined. For St. Jude Directed 6172 electrodes (*n*=4) this was estimated manually based on artifacts generated by the stereotactic marker and stimulation contacts. Finally, VTAs were estimated using a FEM model implemented in Lead-DBS^2^. To validate the electrode reconstruction procedure, we performed a sweet-spot analysis using the VTAs and the DBS-associated differences of task-relevant Parkinsonian motor symptoms according to the right upper extremity subscore of the MDS-UPDRS-III (rUES) scores. First, the VTA center of mass (VTA-CoM) was calculated for each dataset. Starting from a reasonable point close to published sweet-spots for subthalamic DBS for the left side of the brain at MNI coordinates (-13,-13,-6)^6^, we computed the distance for each VTA-CoM to each coordinate in a three-dimensional grid ±4 mm around the published sweetspot. These distances were correlated with the DBS-ON/DBS-OFF differences of the rUES. The sweet-spot would be expected to show the highest negative correlation between the distance to VTA-CoMs and DBS-induced improvements in rUES. The grid search procedure was performed using a resolution of 0.5 mm along the three MNI coordinates followed by a Gaussian smoothing (*sigma=2)* of the resulting 3D matrix in which the minimum was determined as the empirical DBS sweet spot in our dataset.

**Supplementary information 3: Resting-state connectivity analysis**

We used publicly available resting state functional MRI data from young unrelated healthy controls (*n*=100) from the human connectome project. The analysis was performed with custom in-house software written in Matlab and SPM12 as described previously in Klingbeil et al.^7^. All functional datasets consisted of two resting-state sessions (right–left and left–right phase encoding) acquired over 15 minutes with a gradient-echo echo planar imaging sequence with a repetition time of 720 ms and a resolution of 2 mm (isotropic). All images were already partly preprocessed when downloaded, which included gradient distortion correction, motion correction, distortion correction, normalization to MNI space, intensity normalization and bias field removal. Individual VTAs were used as regions of interest (ROIs) to estimate the ROI-to-whole-brain resting-state connectivity as expressed by the Fisher-transformed correlation coefficient between blood-oxygen dependent (BOLD) time series from the VTA voxels and each brain voxel. Signal variance over time explained by nuisance variables (motion parameters, mean white matter, CSF, global signal) was removed using a multiple regression approach. Residual BOLD time series were band-pass filtered (0.01–0.08 Hz), all images with frame-wise displacement >0.5 mm were discarded. Two datasets were excluded because of heavy in-scanner motion. Datasets were smoothed using a Gaussian kernel (full-width at half-maximum 5 mm) after extracting the unsmoothed time-series from the regions of interest to preserve as much information as possible from the small VTA-ROIs. Representative BOLD time-series were expressed as the first eigenvariate of the time series of all voxels within that ROI. ROI-associated networks were calculated based on functional connectivity (i.e., Fisher-transformed Pearson correlation coefficients) between ROI time-series and the time-series of all other brain voxels (“ROI-to-whole brain”). We calculated separate connectivity maps for the left–right and right–left phase encoding session, which were averaged afterwards to obtain a single ROI-to-whole-brain connectivity map for each patient. Then a second-level analysis was performed in SPM12. First, we analyzed which voxels demonstrated a significant connectivity to the VTAs on the group level (p(FWE)<0.05 on the voxel level). In a second step, DBS-related differences in performance gains during training (PI at EOT – BOT), and DBS-induced differences in PD motor symptoms (rUES) were used as regressors. Maps of correlations between these metrics and each voxel connectivity across individual VTA-connectivity maps were then calculated. The R-Maps were broadly compared by calculating voxel-wise correlations between them and the correlations between behavioral metrics and R-Maps were calculated similar to previous research^8^. A leave-one-out approach similar to that employed by Horn and colleagues^9^ was used to assess whether these R-Maps were specific for the behavioral metrics: An R-Map was calculated using the VTA-connectivity maps and behavioral data of all patients except one. Thereafter, the similarity (voxel-wise correlation) of the individual VTA-connectivity map to this R-Map was calculated. This step was repeated for every PD patient. To evaluate the performance of this predictive model, we calculated shared variance (R²) between individual similarity values and the behavioral metric. The analysis was repeated using only voxels which show significant connectivity to the VTAs.

**References**

1. Fedorov, A.*, et al.* 3D Slicer as an image computing platform for the Quantitative Imaging Network. *Magn Reson Imaging* **30**, 1323-1341 (2012).

2. Horn, A.*, et al.* Lead-DBS v2: Towards a comprehensive pipeline for deep brain stimulation imaging. *Neuroimage* **184**, 293-316 (2019).

3. Avants, B.B.*, et al.* A reproducible evaluation of ANTs similarity metric performance in brain image registration. *Neuroimage* **54**, 2033-2044 (2011).

4. Avants, B.B., Epstein, C.L., Grossman, M. & Gee, J.C. Symmetric diffeomorphic image registration with cross-correlation: evaluating automated labeling of elderly and neurodegenerative brain. *Med Image Anal* **12**, 26-41 (2008).

5. Dembek, T.A.*, et al.* Directional DBS leads show large deviations from their intended implantation orientation. *Parkinsonism Relat Disord* **67**, 117-121 (2019).

6. Dembek, T.A.*, et al.* Probabilistic sweet spots predict motor outcome for deep brain stimulation in Parkinson disease. *Ann Neurol* **86**, 527-538 (2019).

7. Klingbeil, J.*, et al.* Pathological laughter and crying: insights from lesion network-symptom-mapping. *Brain* **144**, 3264-3276 (2021).

8. de Almeida Marcelino, A.L., Horn, A., Krause, P., Kuhn, A.A. & Neumann, W.J. Subthalamic neuromodulation improves short-term motor learning in Parkinson's disease. *Brain* **142**, 2198-2206 (2019).

9. Horn, A.*, et al.* Connectivity Predicts deep brain stimulation outcome in Parkinson disease. *Ann Neurol* **82**, 67-78 (2017).
